# Use of the University of Minnesota Biocatalysis/Biodegradation Database for study of microbial degradation

**DOI:** 10.1186/2042-5783-2-1

**Published:** 2012-01-04

**Authors:** Lynda BM Ellis, Lawrence P Wackett

**Affiliations:** 1 Department of Laboratory Medicine and Pathology, University of Minnesota, Minneapolis, MN 55455, USA; 2 BioTechnology Institute, University of Minnesota, St Paul, MN 55108, USA; 3 Department of Biochemistry, Molecular Biology and Biophysics, University of Minnesota, St Paul, MN 55108, USA

**Keywords:** Biodegradation database, pathway prediction, microbial degradation

## Abstract

Microorganisms are ubiquitous on earth and have diverse metabolic transformative capabilities important for environmental biodegradation of chemicals that helps maintain ecosystem and human health. Microbial biodegradative metabolism is the main focus of the University of Minnesota Biocatalysis/Biodegradation Database (UM-BBD). UM-BBD data has also been used to develop a computational metabolic pathway prediction system that can be applied to chemicals for which biodegradation data is currently lacking. The UM-Pathway Prediction System (UM-PPS) relies on metabolic rules that are based on organic functional groups and predicts plausible biodegradative metabolism. The predictions are useful to environmental chemists that look for metabolic intermediates, for regulators looking for potential toxic products, for microbiologists seeking to understand microbial biodegradation, and others with a wide-range of interests.

## Introduction

Microbial degradation here refers to the microbial conversion of organic compounds, often those of that negatively impact human health, to less toxic or more useful forms, in the environment or the laboratory. Knowledge of the genes, enzymes, and pathways involved in this process help us understand environmental processes, obtain useful products, engineer remediation of polluted environments, and predict the fate of chemicals in the environment. This minireview focuses on the past ten years of research in this field; for earlier work, see [1].

Hundreds of thousands of natural products are known; millions of compounds have been synthesized by organic chemists. There is no end in sight to chemical synthesis. The number of stable organic compounds that are less than 500 molecular weight are estimated to range from 10^20 ^to 10^200 ^[2]. Of the chemicals currently sythesized, almost 100,000 are used commercially. Most naturally-occurring molecules, and many of the synthetic ones, are transformed by at least some microbe, somewhere on the earth. How does nature handle such a vast range of compounds?

Life has likely existed on Earth for at least 3.6 billion years, during which time living things have acquired the ability to catabolize almost every available carbon source. It has been estimated that there are 5 × 10^30 ^prokaryotes on Earth [3] and every free-living prokaryote typically contain 1,000 -10,000 genes [4], making for an enzyme diversity of approximately 10^34^. While many of those 10^32 ^enzymes are isofunctional, there is still enormous untapped metabolic diversity in the microbial world. For example, there are a class of antibiotics produced by *Streptomyces *species that contain an azoxy functional group. Until recently [5], there was no information about the enzymes that biosynthesize the azoxy group, and information is still lacking on the catabolism of azoxy groups. There are many examples in the literature of chemical groups identified in natural products for which metabolic information is lacking [1]. Since the structures have been rigorously identified, and the compounds derive from biological sources, novel biosynthetic enzymes must exist. Moreover, since these natural product compounds are not observed to accumulate in the biosphere, it is likely that enzymes participate in their biodegradation.

The range of microbial biodegradative metabolism is broad and, based on the discussion above, expandable almost infinitely. To more systematically organize and display this information reported in the scientific literature, the University of Minnesota Biocatalysis/Biodegradation Database (UM-BBD) [6,7] began in February, 1995.

## Structure of the Biocatalysis/Biodegradation Database

The UM-BBD was developed to compile information on experimentally-determined metabolic pathways that are used by microbes to degrade chemical substances, principally those considered to be environmental pollutants. Metabolic pathways are depicted in text and graphic formats. Pathway representation consists of starting compounds and intermediates and the reactions they undergo. The reactions are typically catalyzed by enzymes although, in some cases, unstable intermediates are generated that undergo spontaneous chemical reactions to produce new intermediates. The enzymes are encoded by genes that are contained within specific microorganisms. The aerobic UM-BBD pathway for the compound toluene [8], highlights these features. In some cases, intermediates, enzymes and genes for some of the pathway may be unknown, as shown for the UM-BBD pathway for isoniazid [9]. The UM-BBD thus represents the current state of knowledge that can spur further studies on pathways it depicts.

**Figure 1:**
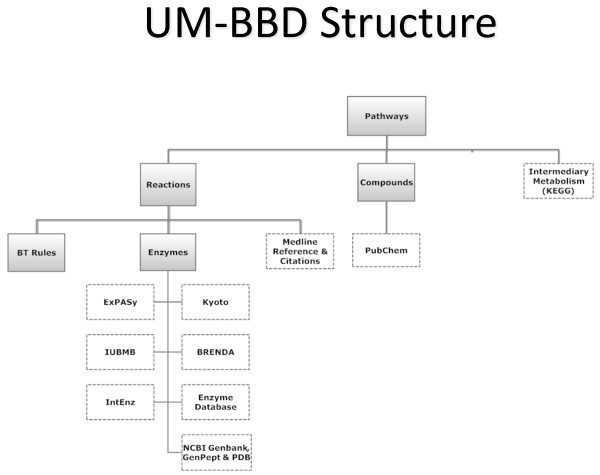
**Structure of the UM-BBD**. A solid box is UM-BBD information; a dotted box is information from one or more links to an external database.

The UM-BBD database structure is shown in Figure 1. This highlights that metabolic pathways are the primary focus and consist of metabolic reactions contained on the UM-BBD server or provided to users via links to sites such as the Intermediary Metabolism database of KEGG [10]. Additional information on enzymes can also be obtained via links from UM-BBD reactions pages to the ExPASy [11], Kyoto [10], or BRENDA [12] databases. Gene sequence data can be obtained by links to the NCBI Genbank [13] database. Most pathways also list microorganisms that have been identified carry out the depicted metabolic pathways, either in full or in part.

The bacteria studied for biodegradative metabolism have most frequently been isolated from soil. Soil bacteria generally have greater transformation capabilites than other bacteria. In soils, food sources are likely to be more limited, diverse, and constantly changing. Bacteria that acquire capabilites to metabolize and grow on a broad range of compounds have a greater advantage. It is believed that more than 99% of bacteria commonly found in soil have never been cultivated in pure culture. This assertion is supported by DNA hybridization studies indicating that 1 g of a non-contaminated soil contained nearly one million prokaryotic species. A heavy metal contaminated soil contained less but still on the order of thousands of species per g soil [14]. Given this enormous diversity, it cannot be definitively asserted which bacterial genera and species are most significant in biodegradation.

Soil microbes that have been isolated for UM-BBD biodegradation pathways are compiled in the University of Minnesota Biocatalysis/Biodegradation Database microorganism list [15]; the most frequently listed genera are *Pseudomonas, Rhodococcus *and *Arthrobacter*, in that order. Environmental fungi can also transform a variety of organic compounds; genera in the UM-BBD include *Aspergillus, Fusarium*, and *Phanerochaete*.

In general, bacteria are more likely to completely degrade, or mineralize, the starting compound whereas fungi may catalyze hydroxylation and functionalization reactions leading to an accumulation of one or more metabolites. For example, fungi metabolize 1-anthrol, a hydroxylated metabolite of anthracene, by functionalizing its hydroxyl group with xylose, glucuronate or sulfate [16]. These functionalization reactions serve to increase the solubility of the metabolite and facilitate its excretion into the aqueous environment surrounding the organism. In some cases, bacteria may also metabolize compounds by functionalization rather than mineralization. For example, bacteria can displace the chloride group in the pesticide alachlor by a thiol-containing molecule, such as glutathione or cysteine [17]. Further enzymatic oxidation of these conjugates produce alachlor-ESA (ethyl sulfonic acid) [18]. Metabolites of alachlor-ESA found in soil and groundwater include alachlor-OA (oxanilic acid) [19] and N-(2,6-diethylphenyl)-ESA [20].

## Reaction Classes Represented by the UM-BBD

The reactions depicted by the UM-BBD capture the wealth of organic functional groups that are transformed by microorganisms. For example, hydrocarbons are oxidized by oxygenase enzymes to produce alcohols, thus transforming a hydrocarbon functionality into an alcohol. The Nomenclature Commission of the International Union of Biochemistry and Molecular Biology (NC-IUBMB) classifies enzyme reactions into 6 major groups, each of which contains many UM-BBD enzymes (Table 1). UM-BBD enzymes are not uniformly represented in the 6 groups, either in NC-IUBMB classification or in the UM-BBD. For example, oxidoreductases are overrepresented, comprising 27% of IUBMB and 62% of UM-BBD enzymes, indicating their importance in all, and even more so in biotransformative, reactions. Each major NC-IUBMB group can be subdivided into three further levels, indicated by sub-codes.

**Table 1:** UM-BBD Enzymes by NC-IUBMB Class

Class	Name	# in UM-BBD
EC 1	Oxidoreductases	552 (62%)
EC 2	Transferases	54 (6%)
EC 3	Hydrolases	144 (16%)
EC 4	Lyases	90 (10%)
EC 5	Isomerases	29 (3%)
EC 6	Ligases	25 (3%)

The UM-BBD details biotransformation reactions for over sixty organic functional groups [21]. A number of the reaction types are quite familiar to all biochemists; those dealing with alcohol, aldehyde, carboxylic acid and approximately thirty other commonly-occurring biological organic functional groups. The UM-BBD also contains information on other chemical groups that may be less familiar; for example, organomercurials, organoarsenicals, alkylboronic acids, and thioamides. It is anticipated that there will be new discoveries of novel metabolic transformations with as yet uninvestigated chemical functional groups. This can be predicted with confidence because the natural product chemistry literature contains references to more than one hundred organic functional groups present in molecules biosynthesized by living things [1]. Since these functional groups are produced, and presumably metabolically degraded, there is a strong indication that metabolic transformation of these structural entities does exist.

## Aerobic vs. Anaerobic Biodegradation

Microbiologists often make a large division in the type of metabolism found in prokaryotes along the lines of aerobic versus anaerobic organisms [22]. As discussed later, this demarcation is not as distinct as often imagined. However, some general principles emerge from dividing metabolism in this way. Generally, microorganisms that live in aerobic environments use oxygen as their final electron acceptor and employ oxygenase enzymes that insert oxygen from atmospheric diatomic oxygen into substrates. As mentioned above, 62% of UM-BBD enzymes are oxidoreductases (EC class 1, Table 1); the largest subgroup within this group are oxygenases.

Strictly anaerobic microorganisms live in the absence of oxygen and thus do not typically have the option to use oxygenases in their metabolism (see an exception to this at the end of this section). This has important consequences for the microbes and the types of biodgradative reactions they carry out. For example, the metabolism of benzenoid aromatic hydrocarbons is completely different when carried out by aerobic or anaerobic bacteria. Aerobic bacteria typically break the aromaticity of the benzene ring and set the substrate up for further oxidation reactions by using dioxygenases to add oxygen to the ring and producing a *cis*-dihydrodiol. Fungi growing in aerobic environments couple monoxygenase and epoxide hydrolase enzymes and make *trans*-dihydrodiols from benzenoid aromatic ring compounds. A search for dihydrodiol compounds on the UM-BBD on February 24, 2011 revealed 27 such compounds that are produced as intermediates in aerobic biodegradation pathways. There is evidence for many additional dihydrodiols being formed in UM-BBD lists of the known reactions catalyzed by naphthalene dioxygenase [23] and toluene dioxygenase [24]. Aromatic rings are subsequently opened into aliphatic compounds by the action of dioxygenases that both add oxygen and cleave a carbon-to-carbon bond in the ring. The ring-opened product is metabolized to intermediates that enter the tricarboxylic acid cycle. An example of such pathways on the UM-BBD is the bacterial aerobic metabolic pathways for toluene [8].

The anaerobic metabolism of benzenoid aromatic compounds proceeds quite differently, as shown in the anaerobic toluene pathway on the UM-BBD [25]. In the anaerobic pathway, the methyl group of toluene is functionalized by benzylsuccinate synthase [26] and further transformed into the metabolite benzoyl-CoA. Benzoyl-CoA is a common intermediate in the anaerobic biodegradation of aromatic ring compounds. In the absence of oxygen, the aromatic ring aromaticity is disrupted by reduction via benzoyl-CoA reductase [27]. Further metabolism produces acetyl-CoA that can be assimilated to make cellular materials or further oxidized for energy.

Aerobic and anaerobic differences in metabolism extend to the BTEX compounds and other molecules. BTEX stands for benzene, toluene, ethylbenzene and xylenes and they are common contaminants found in gasoline spills. The UM-BBD has a metapathway page covering BTEX metabolism. The page highlights the differences in aerobic versus anaerobic benzeonoid ring metabolism [28].

Many reactions are hydrolytic and cannot be ascribed to aerobic or anaerobic organisms exclusively. For example, the atrazine pathway that starts with atrazine dechlorination proceeds through a series of hydrolytic reactions to completely mineralize the *s*-triazine ring. The reactions have been described in aerobic bacteria but there is no metabolic prohibition for anaerobes carrying out a pathway consisting exclusively of hydrolytic reactions. In other cases, reactions are ambiguous and cannot be relegated into neat classes of occurrence in aerobic or anaerobic bacteria. For example, the enzyme acetylene hydratase catalyzes the addition of water to a carbon-carbon triple bond to produce an aldehyde. The enzyme was originally found in a strictly anaerobic bacterium, *Pelobacter acetylenicus*, and it is activated *in vitro *by strongly reducing conditions [29]. Hence it was one time considered to be an enzyme of "anaerobic metabolism." Subsequently, the enzyme activity was identified in aerobic bacteria and purified under both aerobic and anaerobic conditions and obtained in active form *in vitro *[30]. Indeed, enzymes that require strictly anaerobic conditions to maintain activity, such as nitrogenase, are found and remain active in aerobic bacteria, such as *Azotobacter vinelanddi*, even in the presence of a substantial atmospheric oxygen concentration [31].

A more intriguing recent finding is with the anaerobic bacterium *Methylomirabilis oxyfera *[32]. *M. oxyfera *can oxidize methane and respire nitrate. Other anaerobes have been shown to oxidize methane anaerobically via a functional reversal of the methane forming reaction catalyzed by methyl-S-coenzyme M reductase. Surprisingly, *M. oxyfera *used an oxygenase enzyme, which by definition uses gaseous dioxygen, for methane metabolism. The organism generates very low levels of dioxygen from the reduction of nitrate to dinitrogen with the concurrent liberation of dioxygen. These low levels of oxygen are then cleared via the oxygenase-catalyzed oxidation of methane, thus protecting the cell against oxygen toxicity.

A reciprocal observation has been made with *Sterilobacterium denitricans*, a facultative organism that grows aerobically and anaerobically using the steroid ring of testosterone as its sole source of carbon. Testosterone is principally composed of carbon and hydrogen and is typically metabolized via a series of oxygenase-catalyzed reactions. However, *S. denitricans *grows on testosterone by incorporating oxygen from water into the steroid structure and this occurs under both aerobic or aerobic growth conditions [33].

Some reactions that are often considered to be strictly anaerobic are not always restricted to anaerobic bacteria (Table 2). For example, reductive dechlorination is carried out by anaerobic bacteria [34,35] and aerobic bacteria [36] and even oxygenase enzymes [36,37]. The pentachlorophenol catabolic pathway is comprised of oxygen-requiring and reductive dechlorination (third and fourth step) enzymatic reactions [36]. The overall pathway thus requires oxygen and the organism grows aerobically. In another example, it has been shown that an oxygenase, cytochrome P450_cam_, can catalyze reductive dechlorination at a substantial rate, showing a *k_cat_*/K_D _of 2.1 × 10^5 ^L M^-1^s^-1 ^[37]. The reactions proceed under reduced oxygen tension. When oxygen is present, both reductive dechlorination and oxygenative dechlorination reactions can be observed.

**Table 2:** Representative reductive dehalogenation reactions in the UM-BBD having different degrees of information

Substrate	Product	Enzyme, if known	Reference
carbon tetrachloride	chloroform	unknown	[34]
beta-1,2,3,4,5,6-hexa-chlorocyclohexane	delta-1,2,3,4-tetra- chlorocyclohexane	unknown	[35]
tetrachlorohydroquinone	trichlorohydroquinone	tetrachlorohydroquinone reductase	[36]
trichlorohydroquinone	dichlorohydroquinone	trichlorohydroquinone reductase	[36]

## Evolution of Biodegradation Enzymes

Microbes are continually evolving new metabolism in response to new industrial chemicals introduced into the environment [38-40]. The UM-BBD contains many pathways for compounds that have only been synthesized within the last one hundred years, a very short time in evolutionary history. The UM-BBD currently contains approximately 1300 compounds; representative "new" chemicals are shown in Table 3.

**Table 3:** UM-BBD compound names starting with the letter "A" that were first synthesized in the past 100 years and their UM-BBD compIDs

Compound Name	UM-BBD compID
Acetonitrile	c1151
*cis*-Acetylacrylate	c0386
*N*-Acetylisoniazid	c1170
Acrylonitrile	c0148
Ametryn	c0260
Amino(methoxy) sulfanylidenephosphinite	c1480
4-Amino-2-hydroxylamino-6-nitrotoluene	c0442
6-Amino-2-naphthalenesulfonic acid	c0733
2-Aminobenzenesulfonate	c0245
4-Aminobenzenesulfonate	c0551
2'-Aminobiphenyl-2,3-diol	c0445
6-Aminohexanoate trimer	c1000
Aminonitrofen	c1403
Aminoparathion	c0089
Asulam	c1263
Atrazine	c0002

The UM-BBD compound web page for a compound with compID = c1151, for example, is available at: http://umbbd.msi.umn.edu/servlets/pageservlet?ptype=c&compID=c1151

It cannot be definitively ascertained that a chemical compound is not a natural product since it may be produced by some, as yet, uncharacterized organism. However, it seems likely that pesticides like asulam and atrazine are strictly industrial chemicals. Atrazine is now reported to undergo ready biodegradation in many soils [41]. For this to occur, one would have to presume either of two occurences. One hypothesis is that a pre-existing metabolic pathway for something else was able to immediately catabolize atrazine when it first appeared commercially in 1959. The other hypothesis is that pre-existing genes that encoded other reactions were co-opted and evolved new enzyme specificities that allowed them to catabolize atrazine. The latter view is the prevailing paradigm for the evolution catabolic metabolism for atrazine and other anthropogenic compounds [38-40].

The evidence for the recent evolution of atrazine catabolism is multi-faceted. The major pathway for atrazine is intiated by a dechlorination reaction to produce hydroxyatrazine [42]. In early literature, atrazine was considered poorly degradable but fifty years after its introduction it is metabolized moderately to rapidly in many soils [41]. The UM-BBD lists six different bacteria that catabolize atrazine and subsequent reactions in the main degradative pathway. The six bacteria, isolated from several different continents, have been shown to contain isofunctional enzymes that have nearly or completely identical sequences [43]. It is highly unlikely that these enzymes evolved millions of years ago and have not undergone any mutational change when all of the other genes in the different genera have diverged extensively. The atrazine genes were subsequently shown to be localized to broad host range plasmids and flanked by *IS1071 *and *IS1081 *elements that can facilitate their movement amongst populations [44]. Moreover, another study recapitulated the natural evolutionary process in the laboratory [45]. Atrazine dechlorinase was shown to be related to deaminases but did not show deaminase activity. DNA shuffling was conducted with the atrazine dechlorinase gene and a homologous deaminase gene [45]. This produced mutant enzymes with one to five amino acid changes. This showed that only two amino acid changes were necessary to "evolve" a deaminase into a dechlorinase. Moreover, some of the mutant enzymes had new activities not shown by any of the parental enzymes and were active against chemicals which have not yet been studied for microbial degradation. These data illustrated the facility by which microbial enzymes can evolve quickly to handle new chemical entities.

## Predicting Biodegradation Pathways

Only a small fraction of natural and anthropogenic chemicals have been tested with respect to biodegradation. It has been estimated that between 10^20 ^and 10^100 ^stable organic chemical compounds of less than 500 molecular weight are theoretically possible to synthesize [46]. Thus, the gap between the total set of novel chemical substances and those tested for microbial catabolism will continue to increase. This gap will need to be filled by increasing our knowledge of the principles of biodegradation such that we can use the knowledge to predict the fate of chemicals in the environment.

In many fields of science, predictive ability of a larger system is built up from knowledge of its parts and how they are assembled. To predict chemical reactions, one first needs knowledge of the cluster of elements that bond together into stable and defined configurations, the organic functional groups. For example a primary alcohol is drawn with the chemical formula RCH_2_OH where R can represent virtually any organic fragment. All primary alcohols share certain fundamental properties that underlie their reactivity with enzymes or chemical reagents. For example, there are many enzymatic reactions in which a primary alcohol is oxidized to an aldehyde. In another example, it could be phosphorylated. These two examples represent different biotransformations.

By defining all of the functional groups that undergo biotransformation, and all individual reaction types for each, one can define biotransformations in a generalized way. The UM-BBD curators have tried to capture the breadth of known functional group biotransformations. The UM-BBD currently contains information on approximately 70 chemical functional groups. There is a compilation of the majority of functional groups compiled into a Table and a graphic available on the UM-BBD [47]. The UM-BBD depicts multiple types of biotransformations for each functional group; for example, reduction, oxidation, elimination, or hydrolysis). On avergage, there are 3.5 transformations per functional group. This makes for 250 different reactions types overall. By consecutively focusing on different functional groups within a compound, and the reactions they undergo, one can predict a series of biochemical transformations that the compound may plausibly undergo.

This general approach is used by two different publically-available biodegradative pathway prediction systems, the University of Minnesota Pathway Prediction System (UM-PPS [48]) and PathPred [49], both of which are described below.

### University of Minnesota Pathway Prediction System (UM-PPS)

The UM-PPS is a rule-based biodegradation prediction system that was first offered to users in 2002 [50]. It predicts plausible microbial biodegradation pathways that might occur in soil. It predicts both aerobic, anaerobic, and reactions that would occur under aerobic or anaerobic conditions. The UM-PPS is not meant to represent the catabolic pathway(s) of any specific bacterium. Rather it represents catabolic pathways carried out by any bacterium or mixture of bacteria. This latter focus best reflects environmental biodegradation where it is thought that many bacteria often team up to biodegrade anthropogenic chemicals.

**Figure 2:**
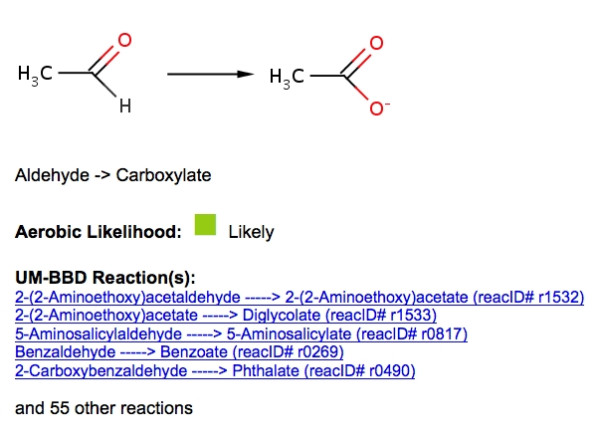
**Example UM-BBD Biotransformation Rule, see text**.

In this context, the UM-PPS brings a constellation of known microbial catabolic reactions to bear on a compound entered by the user to transform it into metabolic intermediates. The transformations are carried out by biotransformation (bt) rules that are based on the known biochemical reactions of functional groups. Based on the discussion in the previous section, it can seen that on the order of 250 biotransformation rules can represent known metabolic reactions. Any general rule thus applied can be linked to one or more known metabolic transformations found in the UM-BBD and/or the scientific literature. For example, rule bt0003 was written for the reaction of an aldehyde to produce a carboxylate (Figure 2). There are 59 reactions in the UM-BBD that correspond to this rule; users can examine those reactions to learn more about the basis of the rule. A few rules, for example the hydrolysis of acyl chlorides to a carboxylic acid, rule bt0026, occur rapidly and spontaneously in water. This reaction has been shown to occur non-enzymatically in biological systems and contribute to the overall biodegradation of certain chlorinated compounds.

**Figure 3:**
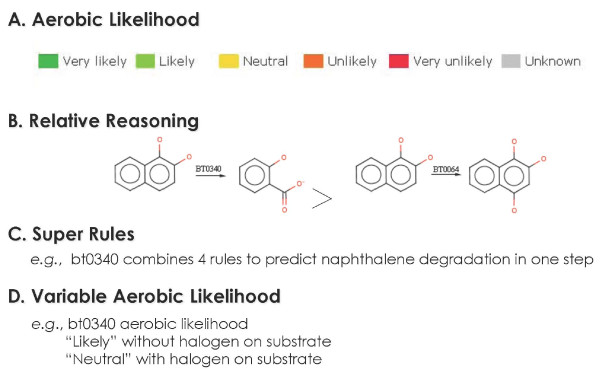
**Different features comprising metabolic logic in the UM-PPS**: A) Each biotransformation rule has an associated, color-coded aerobic likelihood assigned by content experts; B) Relative reasoning is applied when a biotransformation rule take precedence over another; C) Super rules combine metabolic steps when warranted, based on existing biodegradation knowledge; D) Variable aerobic likelihood is used to distinguish the likelihood of rules based on structural characteristics of the compound. For more information on the UM-PPS, see http://umbbd.msi.umn.edu/predict/aboutPPS.html.

Each rule is ranked with respect to its likelihood for occurring in aerobic biodegradation (Figure 3A). In this ranking system, the reactions governed by rules bt003 and bt0026 are considered to be "likely" or "very likely," respectively. During a prediction cycle, each compound submitted to the UM-PPS is examined for the organic functional groups that it contains, and these functional groups are matched to the appropriate UM-PPS rules. There are presently 250 btrules in the system; this number and the individual rules are periodically updated.

When a matching rule is found, the UM-PPS transforms the query molecule containing the given functional group into a product. This process can occur for all the different functional groups of a molecule, so there can be multiple products from a given starting compound. Each product from the first compound can, in turn, be used for further rounds of prediction. A set of consecutive predicted transformations thus constitutes a predicted metabolic pathway. The prediction terminates when the pathway reaches a compound that either cannot be degraded using existing rules or is on a list of termination compounds defined within the UM-PPS. Predictions by default are run for up to six steps (prediction cycles) and are limited to those rules more commonly found in aerobic environments. Users can changes these and other defaults throughout the prediction process.

If btrules alone were used, the predicted metabolites for complex molecules could consist of dozens, even hundreds, of compounds. This is because the rules could be triggered in a different order to produce parallel and interleafing pathways. However, in nature, reactions often proceed in a given order and, by capturing this knowledge, predictions can be improved. In this context, relative reasoning (Figure 3B) was used to prioritize certain rules over others when certain groups were present. For example, during the aerobic catabolism of benzene ring compounds, dioxygenation of the ring and subsequent dehydrogenation occur sequentially without any intervening reactions. Thus, the dehydrogenation rule would have preference over other rules and prevent the depiction of other pathways that have never been observed in nature. Currently, 126 btrules have relative reasoning.

Other ways to limit false predictions include super rules (Figure 3C), rules that encompass several contiguous simple rules that form a small pathway of their own; and variable aerobic likelihood (Figure 3D), that changes the aerobic likelihood of a rule if a given structural feature is present.

The efficacy of the UM-PPS for predicting "real world" metabolism has been evaluated in several recent publications [51-53]. Helbling et al. [52] studied the metabolism of six pharmaceuticals and six pesticides individually spiked into batch reactors seeded with activated sludge. Two-step, aerobic UM-PPS predictions assisted in the indentification of transformation products (TPs) found following HPLC and full-scan mass spectrometry. The 12 compounds produced 26 TPs, 21 of which (81%) were correctly predicted by the UM-PPS. Three more TPs would have been predicted for all twelve compounds if three prediction steps and all (not only common aerobic) rules were used, increasing the successful predictions to 24 (92%). However, those changes would have also increased false positives, and thus were not considered efficient by the authors. The compounds, their PubChem [54] CIDs, number of TPs for each, and number of those TPs correctly predicted after two steps by UM-PPS and PathPred (see below) are shown in Table 4.

**Table 4:** Compound Names, CIDs, TPs, and correct TP predictions by UM-PPS and PathPred (see text)

Name	CID	TPs	UM-PPS	Path-Pred
Atenolol	2249	1	1	1

Bezafibrate	39042	5	4	1

Carbetamide	27689	1	1	0

Clomazone	54778	2	2	2

DEET	4284	2	1	0

Diazepam	3016	2	2	1

Levetiracetam	5284583	1	1	1

Napropamide	27189	2	2	0

Oseltamivir	65028	1	1	0

Propachlor	4931	5	2	0

Tebutam	92299	1	1	0

Valsartan	60846	3	3	0

	**TOTALS**:	26	21	6

An alternative to using the UM-PPS, also studied by Helbling et al. [52], is subtraction of a full-scan high-resolution MS scan taken at t = 0 from the same scan at t > 0, and using the peaks shown only at t > 0 as the candidate list. For 11 of the 12 parent compounds (all except propaclor), the subtractive method generated a higher number of false positives than the UM-PPS.

### PathPred

The PathPred system [55] derives from data in the current Kyoto Encyclopedia of Genes and Genomes (KEGG) [56]. The biodegradation of an entered compound is predicted based on the biodegradation of similar compounds in the KEGG database. That is, the first reaction of the pathway is based on reactions of similar compounds, then the process is repeated for all products of the reactions. These iterations produce the predicted pathway.

While predictions made in this manner will be valid for compounds close in biochemical similarity to KEGG compounds, it will be difficult to predict pathways for dissimilar compounds. Also, when only a part of an entered compound is similar to a KEGG compound, no predictions can be made for the dissimilar part, such as, for example, the heterocyclic ring of diazepam.

When the 12 compounds tested by Helbling et al. [52] were entered into PathPred (version 1.13), with the compound similarity (Simcomp) threshhold set to its lowest value (0.1), only 6 of the 27 known TPs (23%) could be predicted in two steps (Table 4). Presently, PathPred is less suitable than the UM-PPS for predicting biodegradation pathways for pharmaceuticals and pesticides. The PathPred system will improve as more compounds are entered into KEGG and there is more metabolism for the system to map to.

## Conclusions

For 16 years, the UM-BBD has served as a repository for the growing body of knowledge on microbial biodegradative metabolism. KEGG has also expanded to include a substantial amount of biodegradation. However, the vast majority of the millions of known chemicals have not been evaulated for their biodegradation. Computational prediction can help address that problem and the UM-PPS and PathPred are designed to do that. The predictions are useful to government regulatories, industries producing chemicals, and scientists conducting biodegradation research.

## List of Abbreviations

UM-BBD: University of Minnesota Biocatalysis/Biodegradation Database; UM-PPS: University of Minnesota of Minnesota Pathway Prediction System; NC-IUBMB: Nomenclature Commission of the International Union of Biochemistry and Molecular Biology; ESA: ethyl sulfonic acid; BTEX: benzene, toluene, ethylbenzene, and xylenes; TP: transformation product; HPLC: high pressure liquid chromatography; MS: mass spectrometry; KEGG: Kyoto Encyclopedia of Genes and Genomes.

## Competing interests

The authors declare that they have no competing interests.

## Authors' contributions

The two authors are considered joint First Authors. Both authors have read and approved the final manuscript.
